# Treating Pediatric Oncology Patients: The Emerging Role of Radioligand Therapy

**DOI:** 10.3390/cancers17233821

**Published:** 2025-11-28

**Authors:** Theodore W. Laetsch, Lisa J. States, Margot A. Lazow, Aman Chauhan

**Affiliations:** 1 Children’s Hospital of Philadelphia, Philadelphia, PA 19104, USA; laetscht@chop.edu (T.W.L.); states@chop.edu (L.J.S.); 2 Nationwide Children’s Hospital, Columbus, OH 43215, USA; margot.lazow@nationwidechildrens.org; 3 The Ohio State University College of Medicine, Columbus, OH 43210, USA; 4 Sylvester Comprehensive Cancer Center, University of Miami, Miami, FL 33146, USA

**Keywords:** radioligand therapy, pediatric, NETs

## Abstract

Pediatric neuroendocrine tumors (NETs) and neuroblastomas are rare malignancies with poor outcomes when metastatic. Limited treatment options are currently available for pediatric NETs. Recently, radioligand therapy (RLT) consisting of a radionuclide attached to a ligand, such as [^177^Lu]Lu-DOTA-TATE, has been approved for the treatment of NETs in adolescents aged ≥12 years. Although long-term safety of RLT in adolescents and other pediatric patients needs to be further investigated, data from large adult studies and early pediatric studies suggest feasibility and low toxicity. Future research is needed to assess potential combinations of RLTs with conventional chemotherapy and radiation sensitizers in order to optimize the treatment for pediatric patients with NETs. This review highlights the current status and future directions for RLTs as theranostics for pediatric patients with NETs and neuroblastomas.

## Simple Summary

Radioligand therapies (RLTs), that consist of radionuclides that target specific markers associated with certain cancer cells, are an emerging treatment option for pediatric patients with hard-to-treat diseases like neuroendocrine tumors or neuroblastomas. Recent data from adult and early pediatric studies in the literature indicate that RLT has low toxicity and the potential for efficacy in these patients. Herein, we explore the role of radioligands in imaging and theranostics and the rationale for their use in children and adolescents. Future directions include exploring new biomarkers and the potential for combination approaches with RLT.

## 1. Introduction

Neuroendocrine tumors (NETs) are a heterogeneous group of neoplasms, arising from secretory cells of the neuroendocrine system [1]. In neuroblastoma, the catecholamine biosynthetic and secretory pathways are poorly developed [2]. It is the most common pediatric extracranial solid malignancy, responsible for 10% of all childhood cancers and for approximately 15% of all cancer deaths in children [3]. The incidence rate of neuroblastoma is 11–13 per 1,000,000 in children under 15 years of age [4]. Neuroblastomas can occur anywhere along the sympathetic chain, with the adrenal glands being the most frequently affected area [3,5].

Other NETs, including gastroenteropancreatic NETs (GEP-NETs) and pheochromocytomas and paragangliomas (PPGLs), characterized by more mature catecholamine biosynthetic and secretory pathways, are rare in children, with an incidence of 0.5–2.8 per 1,000,000 people [2,6,7]. Outside of neuroblastomas, GEP-NETs are the most prevalent pediatric NETs [6]. The appendix is the most common location of NETs in children; however, early-stage appendiceal NETs exhibit a benign clinical course [8,9,10]. Recurrence and metastasis are more common with extra-appendiceal tumors [10]. PPGLs are tumors arising from chromaffin cells of the adrenal gland (pheochromocytomas) and extra-adrenal tissues (paragangliomas); approximately 2% of cases of pediatric hypertension are due to PPGLs [11,12]. Especially in children, these tumors are frequently associated with cancer predisposition syndromes.

In this review article, we discuss the recent literature related to the use of radioligand therapies (RLTs) as theranostics for pediatric patients with NETs and neuroblastomas. Articles were initially identified through PubMed literature searches from 2019 to 2024 for radionuclide therapy, pediatrics, and treatment.

## 2. Current Use of Radionuclide Therapies in Pediatric Patients with Neuroblastoma or NETs

### 2.1. Radionuclide Therapies Overview

**Figure 1:**
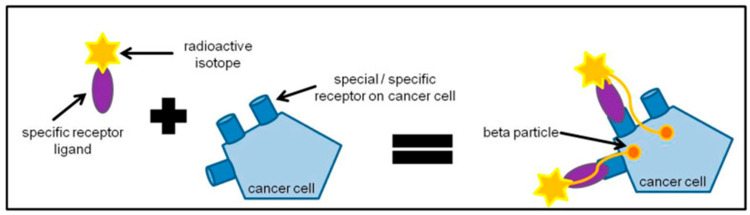
General principles of radioligand therapy. Tumor cells express specific receptors on their cell surface. Radiopharmaceuticals can bind those specific targets and deliver therapeutic doses of radiation to the tumor within close proximity and circumventing radiation exposure to healthy tissue.

Radionuclide therapy, or molecular radiotherapy, consists of the systemic or locoregional administration of a radiopharmaceutical, comprising a radionuclide attached to a ligand, such as an antibody, peptide, or small molecule, via a chelator (Figure 1) [13,14]. Radiopharmaceuticals can bind to specific targets that are overexpressed in the tumor cells, delivering therapeutic doses of radiation preferentially to the tumor while circumventing radiation exposure to healthy tissue [15]. Hence radionuclide therapy has the potential to target cancer cells more specifically compared with external beam radiation therapy [13,14]. Radionuclide therapy has been used for the treatment of NETs for several decades, primarily using β-particle emitters, such as ^131^I, ^90^Y, or ^177^Lu. Recently, highly potent α-particle emitters (e.g., ^225^Ac, ^212^Pb, and ^211^At) have emerged and are under investigation in preclinical, phase 2, and randomized phase 3 studies [13,14].

### 2.2. Radionuclides in Imaging and Theranostics

**Figure 2:**
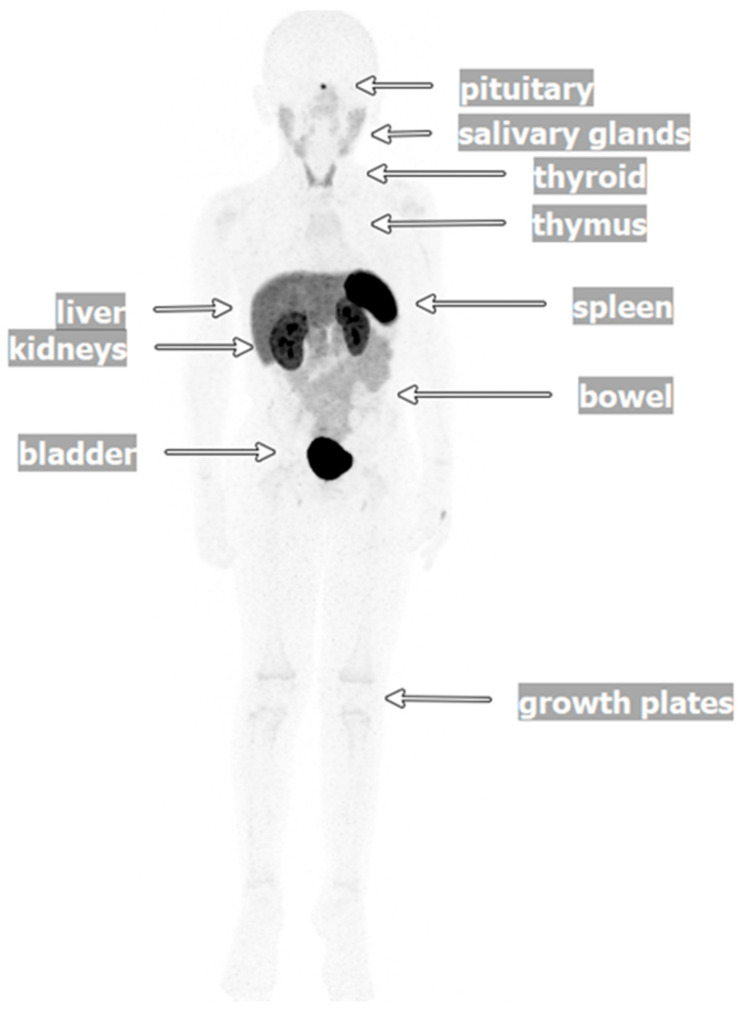
Normal ^68^Ga-labeled DOTA-D-Phe^1^-Tyr^3^-Thr^8^-octreotate (^68^Ga-DOTATATE) imaging in a pediatric patient. Imaging of a normal ^68^Ga-DOTATATE scan in a 5-year-old patient. This three-dimensional maximum-intensity projection image shows normal physiologic uptake in the pituitary gland, salivary glands, thyroid, thymus, liver, spleen, kidneys, bladder, bowel, and long bone growth plates.

**Figure 3:**
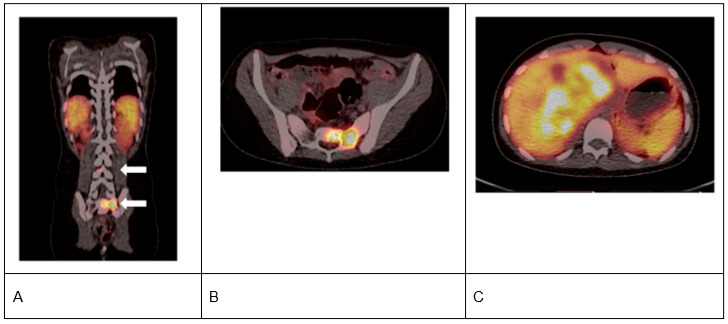
**^68^Ga-DOTATATE imaging in a pediatric patient with pheochromocytoma**. Illustration of somatostatin receptor (SSTR)-positive metastatic pheochromocytoma in a 12-year-old female. (**A**) (coronal fused positron emission tomography [PET]/computed tomography [CT] image) and (**B**) (transverse fused PET/CT image) show SSTR-positive disease in sacrum and lumbar spine (arrows). (**C**) illustrates SSTR-positive hepatic metastatic disease.

**Figure 4:**
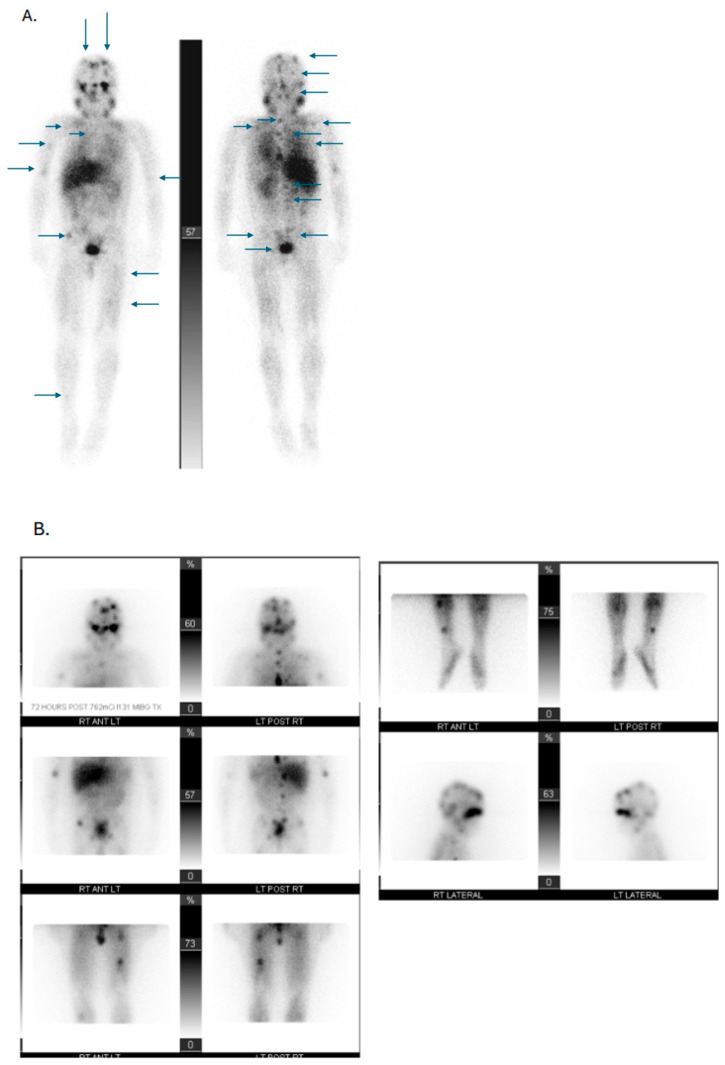

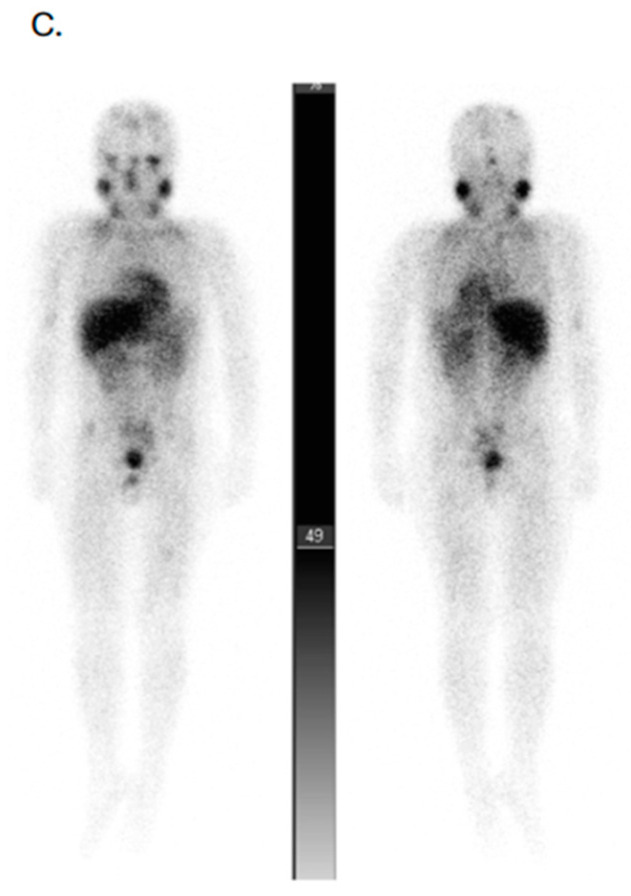
^123/131^I-meta iodobenzyl-guanine (^123/131^I-MIBG) imaging in pediatric patients. (**A**): Diagnostic ^123^I-MIBG scan. Frontal anterior and posterior planar images show multiple foci of uptake (as indicated by arrows) including the skull and skull base, cervical and thoracic spine, sternum, ribs, right clavicle, lumbosacral spine, pelvic bones, bilateral humeri, left femur, and right tibia. (**B**): ^131^I-MIBG therapy scan performed 6 days after therapy infusion. The images illustrate the anterior and posterior body and right lateral and left lateral head planar images and show similar distribution of multiple foci of uptake in the calvarium, occiput, right clavicle, scapula, sternum, ribs, bilateral humeri, cervical, thoracic, and lumbosacral spine, pelvic bones, left femur, and right tibia. Uptake is now seen in the right proximal femur. Note that the radiotracer activity is more pronounced in the skull base, occiput, left femur, and lower sternum than on the diagnostic scan. (**C**): Diagnostic ^123^I-MIBG scan performed 6 months after pre-therapy scan and after two ^131^I-MIBG therapy infusions. The scan shows a partial response to therapy with decreased radiotracer uptake in the skull including skull base, lumbosacral spine, pelvis, bilateral humeri, scapulae, right clavicle, and left femur, and resolution in the right tibia, right femur, cervical and upper thoracic spine, sternum, and ribs.

Radiopharmaceuticals are used as theranostics (i.e., therapy and diagnostics), often leveraging different doses and/or radioisotopes, known as theranostic pairs, to aid in diagnosis, staging, and targeted treatment [16,17,18]. Over the past decade, radiopharmaceuticals such as ^68^Ga-labeled DOTA-D-Phe^1^-Tyr^3^-Thr^8^-octreotate (^68^Ga-DOTATATE), [^177^Lu]Lu-DOTA-TATE (^177^Lu-DOTATATE), and ^123/131^I-meta iodobenzyl-guanine (^123/131^I-MIBG) have been routinely utilized for molecular imaging and subsequent therapy in adults and children (Figure 2, Figure 3 and Figure 4) [16,19,20,21]. The somatostatin receptors (SSTRs) are successful targets for which theranostics were developed [16]. Somatostatin is the natural ligand to SSTR-2A, and somatostatin analogs (SSAs) have been developed to inhibit NET growth [16]. SSTRs regulate cell growth through complex downstream modulation of both proliferation (mitogen-activated protein kinase, protein tyrosine phosphatase) and apoptosis signaling pathways, therefore representing a potential therapeutic target in pediatric oncology [22,23,24]. SSTR-2A expression, in particular, has been reported in pediatric neuroblastomas, GEP-NETs, and PPGLs [7,25,26,27]. Radiolabeling SSAs led to the development of RLT.

The radioligand ^68^Ga-DOTATATE is used as a tracer for positron emission tomography (PET), with a sensitivity of 83–88% and a specificity of 80–100% in NETs [20,28]. ^68^Ga-DOTATATE PET/computed tomography (CT) has demonstrated excellent sensitivity (~92%) in the detection of PPGLs in pediatric and adult settings (Figure 2 and Figure 3) [18,29]. Uptake of ^68^Ga-DOTATATE has been reported in the pituitary gland, spleen, urinary tract, kidney, adrenal glands, uncinate process of pancreas, and liver, with mild homogenous intake observed in the thyroid gland and salivary glands, including the parotid [30,31,32]. Other minimal-to-mild uptake of ^68^Ga-DOTATATE has been detected in thymus, muscles, bones, breast, lungs, and mediastinum in patients with known or suspected neuroendocrine malignancies (Figure 2) [33]. In a dosimetry study, the measured dosimetry of ^68^Ga-DOTATATE was similar to or slightly greater than those for other ^68^Ga-labeled SSAs, such as ^68^Ga-[tetraxetan-D-Phe^1^, Tyr^3^]-octreotide (^68^Ga-DOTATOC) and [^68^Ga-DOTA,1-Nal^3^]-octreotide (^68^Ga-DOTANOC) [31]. The organ doses and effective doses were similar for ^68^Ga-DOTATATE and ^68^Ga-DOTATOC, while the dosimetry for ^68^Ga-DOTANOC was the lowest [31]. In order to predict tissue distribution of ^68^Ga-DOTATATE, a physiologically based pharmacokinetic model was developed in patients without detectable NETs [34]. Tissue distribution, determined by variability in SSTR2 expression, revealed high inter-individual variability in SSTR2 density, also observed when assessing ^68^Ga-DOTATATE PET/CT.

^177^Lu-DOTATATE is a U.S. Food and Drug Administration (FDA)-approved therapeutic agent for the treatment of SSTR-2A-positive tumors [21,35]. The ^177^Lu-DOTATATE mechanism of action has been described previously by Hennrich and Kopka, 2019 [36].

MIBG is a catecholamine precursor that is incorporated within NET cells of neuroblastomas and PPGLs [18]. ^123^I-MIBG scintigraphy shows high specificity (83–100%) and sensitivity (88–93%) and is recommended for neuroblastoma staging, therapeutic response monitoring prognostication, and determining eligibility for ^131^I-MIBG (Figure 4) [37]. However, ^123^I-MIBG imaging has low spatial resolution and about 10% of neuroblastomas do not localize ^123^I-MIBG [38,39]. Magnetic resonance imaging and contrast-enhanced CT are recommended for diagnostics, staging, and identifying image-defined risk factors [37]. While some medical centers perform whole-body single-photon emission-CT (SPECT) scans at every visit for ^123^I-MIBG imaging, there is currently no standard practice for neuroblastoma imaging. For MIBG non-avid tumors, ^18^F-fluorodeoxyglucose PET/CT (^18^F-FDG PET/CT) is recommended but may not be readily available in under resourced regions [37]. ^68^Ga-DOTATATE can also be used for neuroblastoma imaging, and a recent study has suggested that, as ^68^Ga-DOTATATE and ^123^I-MIBG scans yield complementary information, the use of both ^68^Ga-DOTATATE and ^123^I-MIBG imaging scans could be considered [40]. Current challenges regarding the development of theranostics in pediatric oncology include a limited patient population and a wide variety of tumor types, which may require different targets [19].

### 2.3. Mechanism of RLTs and Rationale for Their Use in Children

There are limited therapies available for children with unresectable non-neuroblastoma NETs, and the outcomes for patients with high-risk neuroblastoma remain suboptimal despite intense therapy. With the development of radiopharmaceuticals and theranostics in adult oncology settings, further investigations regarding the potential to translate and apply these in pediatric settings are needed [16]. While some targets are shared across pediatric and adult cancers (such as SSTR-2A expression in NETs), other pediatric cancers have unique molecular targets that are not expressed in adult cancers, providing additional opportunities for the diagnosis and treatment of pediatric cancers [16]. Ganglioside GD2 (GD2) is a glycosphingolipid expressed in tumor cells in neuroblastomas, osteosarcomas, and glioblastomas and is a promising tumor-associated antigen for targeted immunotherapy [16,41,42]. Dinutuximab (Unituxin^®^, United Therapeutics, Silver Spring, MD, USA) is a monoclonal antibody binding GD2 that received FDA approval as part of first-line therapy for children with high-risk neuroblastoma, and dinutuximab beta (Qarziba^®^, EUSAPharma, Hemel Hempstead, UK) has been approved by the European Medicines Agency (EMA) for the treatment of GD2-positive neuroblastoma tumors in children [43,44]. Radiopharmaceuticals, such as ^64^Cu-labeled hu14.18K322A and [^89^Zr] dinutuximab, have been developed to image and/or treat GD2-positive tumors [45,46]. Another mechanism for targeting pediatric cancerous cells is through B7-H3 (cluster of differentiation 276), which is a membrane protein expressed on antigen-presenting cells and present in pediatric gliomas and neuroblastomas [16,47]. Omburtamab, a murine antibody, has a high affinity for B7-H3 and when combined with ^124^I or ^131^I becomes a theranostic agent used for tumor imaging [48].

The role of imaging in the management of NETs is not well established in pediatric settings. Imaging recommendations for adults include CT, magnetic resonance imaging (MRI), SSTR-sensitive imaging, and ultrasound; however, further investigation regarding imaging approaches in pediatric patients is needed [49]. A retrospective study (October 2008 to January 2012) of ^68^Ga-DOTATATE PET/CT and RLT, including ^177^Lu-DOTATATE, ^111^In-DOTA-octreotate (^111^In-DOTATATE), and ^90^Y-DOTA^0^-Tyr^3^-octreotide (^90^Y-DOTATOC), therapies in 14 pediatric patients showed that ^68^Ga-DOTATATE PET was positive in many of these patients, overall correlating with SSTR2 detected by immunohistochemistry [50]. Compared with MIBG imaging, additional sites of disease were visualized with the ^68^Ga-DOTATATE PET, which suggested that ^68^Ga-DOTATATE PET/CT could become a useful molecular imaging technique for pediatric patients with neuroblastoma [50]. ^68^Ga-DOTATATE PET imaging identified bone lesions in 97% and soft-tissue lesions in 100% of pediatric patients [40]. However, MRI remains the conventional imaging test of choice for pediatric patients, if available, due to the lack of ionizing radiation and high contrast and spatial resolution.

## 3. Efficacy of Current RLTs Used for Neuroblastoma and NETs in Pediatric Settings

While the efficacy and safety of ^131^I-MIBG has been recently studied for children with neuroblastoma [51], studies evaluating the efficacy of other RLTs in children are very limited. Current ongoing trials using RLT are summarized in Table 1. These include both focused pediatric trials and trials primarily for adult patients that extend the lower age limit to include adolescents. Pediatric enrollment in the later trials may be very limited in some cases. A recent study evaluated the diagnostic performance and clinical efficacy of ^68^Ga-DOTATATE PET/CT and ^177^Lu-DOTATATE combined with chemotherapy in 14 children with relapsed or refractory metastatic neuroblastoma [52]. Of the five patients who underwent RLT, two showed an initial complete response, which relapsed a few months later, one patient had a partial response, and two had progressive disease [52]. A phase 2, open-label, multicenter, single-arm trial of ^177^Lu-DOTATATE in pediatric patients with primary refractory or relapsed high-risk neuroblastoma is currently ongoing (LuDO-N; NCT04903899) [53]. The main objective of this study is to assess the response to ^177^Lu-DOTATATE treatment in these patients. Secondary objectives include assessment of long-term survival and response and treatment-related toxicity. Another phase 1/2, open-label study of ^177^Lu-DOTATATE in pediatric patients with recurrent and/or progressive high-grade central nervous system (CNS) tumors and meningiomas is currently ongoing (NCT05278208) [54]. Primary endpoints of the pediatric (phase 1) cohort are to estimate the maximum tolerated dose and the recommended phase 2 dose of ^177^Lu-DOTATATE, to calculate the incidence of treatment-related adverse events (AEs), and to assess the efficacy of ^177^Lu-DOTATATE in these CNS-tumor patients evaluated through progression-free survival (PFS). Other endpoints include response rate and antitumor activity of ^177^Lu-DOTATATE, prevalence of SSTR-2A expression, and correlation with clinical and molecular features in this pediatric and young adult neuro-oncology population [54].

Another phase 2 study reported efficacy of ^177^Lu-DOTATATE in patients aged 11–87 years with inoperable, well-to-moderately differentiated, metastatic NETs [55]. However, very few of these patients were children (mean age: 58.5 years). Median PFS for all patients was 11.2 months (337.4 days; range 1–1070 days) [55]. Average PFS for evaluable patients (*n* = 69, 52.27%) who were alive at data cutoff and demonstrated and maintained favorable response (stable disease and partial response) was 31.9 months (range 15.8–68.6 months). Among the 132 evaluable patients, 12 patients achieved a partial response, 66 patients had stable disease, and 54 patients had progressive disease [55]. The objective response rate for the patients who completed treatment, which included complete response and partial response, was 9.09% (*n* = 12) with a cumulative disease control rate (complete response, partial response, and stable disease) of 59.09% (*n* = 78). Of the 28 patients who completed ^177^Lu-DOTATATE therapy (4 or more cycles of treatment), the majority showed disease control (85.71%, *n* = 24) either as partial response (*n* = 8) or stable disease (*n* = 16) [55]. The objective response rate for patients who completed treatment was 28.57% (*n* = 8), and four patients experienced progressive disease. However, pediatric-specific outcomes were not reported.

## 4. Safety of Current RLTs Used for Neuroblastoma and NETs in Pediatric Settings

Currently, safety data of RLTs in pediatric settings are more limited than adult settings. In adult settings, the phase 3, open-label, randomized controlled NETTER-1 trial reported the long-term safety of ^177^Lu-DOTATATE in combination with best supportive care versus best supportive care alone in adult patients with metastasized or locally advanced midgut NETs [65]. No new safety signals were reported during the long-term follow-up analysis (5 years after the last patient was randomized). Treatment-related serious AEs of Grade ≥3 were reported in 3% of patients in the ^177^Lu-DOTATATE in combination with best supportive care arm. In the ^177^Lu-DOTATATE in combination with best supportive care arm, 2% of patients developed myelodysplastic syndrome, of whom one died 33 months after randomization and was the only treatment-related death reported [65,66]. No cases of acute myeloid leukemia have been reported [65].

A real-world study reported long-term efficacy, survival, and safety of ^177^Lu-DOTATATE in 1214 adult patients with bronchial- and GEP-NETs from the ERASMUS Medical Center in Rotterdam, the Netherlands [67]. Long-term toxicities included acute leukemia reported in four (0.7%) patients and myelodysplastic syndrome in nine (1.5%) patients. No therapy-related long-term renal or hepatic failures were reported.

A study evaluated pituitary function in 68 adult patients with NETs who received ^177^Lu-DOTATATE (7.4 GBq/m^2^/cycle, 10 ± 2 weeks apart, for up to 9 cycles) [68]. The median follow-up from inclusion was 30 months. No cases of severe endocrinopathy were detected, and no significant deficiencies in thyroid or adrenal axes were observed, but a decrease in insulin-like growth factor 1 (IGF1) levels was identified [68]. This decrease was dependent on the number of ^177^Lu-DOTATATE cycles received, with no clinically meaningful decrease in IGF1 levels until cycles 7–9. Additionally, most of the patients had received SSA therapy prior to ^177^Lu-DOTATATE, which is known to inhibit growth hormone (GH) secretion, and thus their effect may have contributed to decreased GH/insulin-like growth factor 1 axis function as well. Given the potential risk of endocrinopathy and GH/IGF1 axis dysfunction, close monitoring of endocrine function, including IGF1 levels, should be considered especially in young, skeletally immature patients.

Unlike adult patient studies of RLTs, existing pediatric safety data with RLTs are largely limited to short-term toxicities; long-term safety profiles of these therapies are yet to be investigated. Current ongoing trials using RLTs are summarized in Table 1.

A phase 2 study reported safety results of ^177^Lu-DOTATATE in patients, including some pediatric patients (mean age: 58.5 years; range 11–87 years), with inoperable, well-to-moderately differentiated, metastatic NETs [55]. These safety data reported that 4 cycles of ^177^Lu-DOTATATE administered at intervals of 6 to 9 weeks were effective in tumor burden reduction and had a good safety profile. Abdominal pain, diarrhea, flushing, and fatigue improved in more than half of the patients, and weight loss improved in almost a third of the patients. No Grade 3 or 4 renal toxicities were reported. Grade 3 and Grade 4 hematologic toxicities were reported in 11/132 patients and 5/132 patients, respectively. Grade 3 hepatotoxicity was observed in 3/132 patients [55].

A phase 1 trial of ^90^Y-DOTATOC therapy in 17 children and young adults with refractory SSTR-positive solid tumors, including neuroblastoma and paraganglioma, determined the dose/toxicity profile of this therapy [61]. ^90^Y-DOTATOC was administered in 3 cycles, 6 weeks apart, starting from 1.11 GBq/m^2^/cycle and increasing to 1.85 GBq/m^2^/cycle. No dose-limiting toxicities were observed. Overall, ^90^Y-DOTATOC therapy reported a good safety profile in these patients. The following phase 2 prospective study of ^90^Y-DOTATOC dosimetry in pediatric and young adults with NETs reported that the renal dosimetry was feasible using ^90^Y-DOTATOC time-of-flight PET/CT and bremsstrahlung SPECT/CT [69].

The safety of ^68^Ga-DOTATATE PET/CT and ^177^Lu-DOTATATE, ^111^In-DOTATATE, and ^90^Y-DOTATOC therapies have been retrospectively investigated in six pediatric patients with refractory metastatic neuroblastoma [50]. No significant toxicities were attributed to RLTs. Two patients with baseline thrombocytopenia due to chemotherapy and/or ^123/131^I-MIBG developed Grade ≥3 thrombocytopenia following ^177^Lu-DOTATATE alone and in combination with ^111^In-DOTATATE, respectively. One patient developed pancytopenia with ^177^Lu/^90^Y-DOTATATE combination after 7 cycles of RLT, although attribution was challenging given concurrent/ongoing chemotherapy. The study suggested that RLTs were feasible and well tolerated, with responses observed in patients with progression despite multimodality treatment [50]. These data supported ongoing clinical trials in such patients.

Recently, ^177^Lu-DOTATATE has been approved by the FDA for the treatment of pediatric patients 12 years and older with SSTR-positive GEP-NETs, including foregut, midgut, and hindgut NETs, on the basis of data from the phase 2 NETTER-P trial [21]. The NETTER-P trial evaluated the safety of ^177^Lu-DOTATATE in pediatric patients with SSTR-positive, well-differentiated, Grade 1–2 GEP-NETs or PPGL (NCT04711135) [58]. During cycle 1 of ^177^Lu-DOTATATE, AEs were reported in 10/11 (91%) patients [58]. The most common AEs in cycle 1 were lymphopenia/lymphocyte count decrease and headache, each reported in 4/11 (36%) patients. Grade ≥3 AEs were observed in 4/11 (36%) patients; the most common was lymphopenia/lymphocyte count decrease (2/11; 18%). Overall, during the treatment period, all patients experienced at least one AE; the most common was lymphopenia/lymphocyte count decrease (7/11; 64%). Grade ≥3 AEs related to ^177^Lu-DOTATATE were reported in 5/11 (45%) patients; the most common were lymphopenia/lymphocyte count decrease (5/11; 45%) and neutropenia/neutrophil count decrease (3/11; 27%). There were no ^177^Lu-DOTATATE discontinuations related to AEs and no treatment-related nephrotoxicity observed. No new safety signals were identified in adolescents, and the safety profile was consistent with that of adults. However, it should be noted that safety was only evaluated in nine pediatric patients.

## 5. Future of RLT in Pediatric Settings

SSTR overexpression is found in many pediatric tumors including neuroblastomas and NETs, making it a strong target for RLT; however, other potential biomarkers are currently under investigation as RLT targets, such as integrin, fibroblast activation protein, and delta-like ligand 3 [70,71,72]. Although long-term safety of RLT in these patients needs to be further investigated, data from large adult studies and early pediatric studies suggest feasibility and low toxicity. Currently, highly potent α-particles emitters are being investigated in adult populations and might be of use in pediatric settings in the future [13,14]. Furthermore, the radionuclide ^64/67^CuCl_2_ is currently being investigated as a PET imaging probe in nuclear medicine clinics for cancers, and MeCO-Sar (5-(8-methyl-3,6,10,13,16,19-hexaaza-bicyclo [6.6.6]icosan-1-ylamino)-5-oxopentanoic acid), conjugated to (Tyr^3^)-octreotate (^64^Cu-CuSarTATE), could be of interest for the detection and treatment of SSTR2-positive NETs in the future [73,74,75]. Future research is needed to assess RLT in other histologies besides GEP-NETs, and research on potential combinations of RLT with conventional chemotherapy, DNA repair inhibitors (DNA-dependent protein kinase inhibitors, poly-ADP-ribose polymerase inhibitors), radiation sensitizers (ribonucleotide reductase inhibitor), and/or with oral-targeted therapies (cabozantinib, sunitinib) could maximize DNA damage/tumor control, especially for aggressive relapsed tumors that likely require multi-agent, multimodal therapy [76].

Additionally, there are many ethical and regulatory challenges that are unique to pediatric research. For example, preclinical data in pediatric diseases and safety data in juvenile animals is often insufficient [77]. The types of cancers occurring in children are often different biologically and molecularly than those in adults, and the rarity of pediatric cancer limits potential market size and therefore financial incentives for pediatric-specific drug development. Pediatric patients are therefore often excluded from clinical trials, with pediatric drug development lagging an average of 6.5 years behind that in adults [78]. While recent FDA and EMA guidance has increased the number of trials that include both children and adults [79], often allowing inclusion of patients 12 years and older at adult dosing, some trials are insufficiently adapted for pediatric patients and/or not opened at centers that care for a pediatric population, limiting enrollment of children [80]. Furthermore, dosing for pediatric patients often should be scaled by body surface area (relative to adult dosing) for optimal dosimetry and mitigating toxicity, which deserves further study in the radioligand field. Pediatric patients are also more sensitive to ionizing radiation, with radiation therapy increasing the risk of second primary malignancies [81]. Pediatric trials thus should ensure patients are followed for long periods of time (>10 years) due to long radiation latency periods [81].

## 6. Conclusions and Future Directions

Neuroblastomas, GEP-NETs, and PPGLs are the most common NETs in children. Novel therapies are necessary to improve outcomes for patients. Over the past decade, radiopharmaceuticals, such as ^68^Ga-DOTATATE, ^177^Lu-DOTATATE, and ^123/131^I-MIBG, have been utilized for molecular imaging and treatment in adult and pediatric settings. Outside of the use of ^123/131^I-MIBG for neuroblastoma, these theranostics have been investigated mainly in adults; hence, further investigations regarding the potential to translate and apply these in pediatric settings are needed. Results from pediatric studies, such as the NETTER-P trial in pediatric patients with GEP-NETs and PPGLs, could be promising for the future development of RLTs in pediatric patients with NETs. Theranostics holds immense promise in pediatric oncology, offering the potential to deliver highly targeted, image-guided therapy with minimized systemic toxicity—an approach particularly valuable in children, where treatment precision may directly translate to long-term survivorship and quality of life.

## Figures and Tables

**Table 1:** Summary of pediatric oncology trials using RLTs.

Study	Study Design	Patient Population	N	Endpoints	Key Results
Studies in pediatric populations only					
Retrospective [52]	^177^Lu-DOTATATE combined with chemotherapy	Pediatric patients (age: 4–9 years) with SSTR-positive relapsed/refractory metastatic neuroblastoma	14	Safety Toxicities OS	**Efficacy:** 5 patients underwent RLT: 2 had CR, which relapsed a few months later, 1 showed PR, and 2 showed PD OS estimated at 14.5 months (95% CI, 8.9–20.1) **Safety:** Leukopenia: Grade 1 and Grade 2 reported in 4 and 1 patients, respectively Anemia: Grade 1 and Grade 2 reported in 2 patients each Thrombocytopenia: Grade 1 and Grade 2 reported in 2 and 3 patients, respectively Grade 1 serum creatinine reported in 1 patient
Phase 2, open-label, multicenter, single-arm trial (LuDO-N; NCT04903899) [53]	^177^Lu-DOTATATE	Pediatric patients with relapsed/refractory high-risk neuroblastoma	Recruiting	To assess response to ^177^Lu-DOTATATE single agent by RECIST 1.1 at 1 and 4 months after completion of therapy PFS OS Hematologic and renal toxicity per CTCAE 5.0	Not published yet
Phase 1/2, open-label trial (NCT05278208) [54]	^177^Lu-DOTATATE	Pediatric patients with progressive/recurrent high-grade CNS tumors and meningiomas	Recruiting	MTD RP2D TRAE by CTCAE 5.0 PFS ORR Antitumor activity of ^177^Lu-DOTATATE Prevalence of SSTR-2A expression and correlation with clinical and molecular features	Not published yet
Phase 1, open-label trial (NEUROBLU 02; NCT03966651) [55]	^177^Lu-DOTATATE	Pediatric patients with SSTR-positive refractory/recurrent neuroblastoma	Recruiting	MTD Safety	Not published yet
Phase 2, open-label trial (LUPARPED; NCT06607692) [56]	^177^Lu-DOTATATE combined with olaparib	Pediatric patients with SSTR-positive recurrent/relapsed solid tumors	Recruiting	ORR	Not published yet
Phase 2, NETTER-P trial (NCT04711135) [57]	^177^Lu-DOTATATE	Pediatric patients, aged 13–17 years with SSTR-positive GEP-NETs or PPGL	11	Absorbed radiation dose in target organ Safety Toxicities PK	**Dosimetry:** Median (range) cumulative administered activity was 28.2 (7.3–29.9) GBq The projected median cumulative absorbed doses for four administrations were 21 (range: 14–40) Gy in kidneys and 0.76 (range: 0.55–1.00) Gy in bone marrow, based on blood data; these dosimetry values were predicted to be within safety thresholds for adolescents and adults **Safety:** The most common AEs were lymphopenia/lymphocyte count decrease and headache reported in 4/11 (36%) patients, each, during cycle 1 of ^177^Lu-DOTATATE Grade ≥3 AEs occurred in 4/11 (36%) patients during cycle 1 of ^177^Lu-DOTATATE During the treatment period, the most common AE was lymphopenia/lymphocyte count decrease, reported in 7/11 (64%) patients Grade ≥3 AEs related to ^177^Lu-DOTATATE during treatment period were reported in 5/11 (45%) patients; the most common were lymphopenia/lymphocyte count decrease (5/11; 45%) and neutropenia/neutrophil count decrease (3/11; 27%) No treatment-related nephrotoxicities were observed
**Studies in populations of young adult or pediatric patients**					
Phase 2, open-label, diagnostic study (NCT04559217) [58]	^68^Ga-DOTATATE and ^123^I-MIBG	Patients (≤21 years) with neuroblastoma	Recruiting	Accrual rate Rate of AEs Positive lesions for ^68^Ga-DOTATATE Discordance of positive lesions for ^68^Ga-DOTATATE and positive lesions of ^123^I-MIBG	Not published yet
Phase 1 (NCT00049023) [59]	^90^Y-DOTATOC and co-administration of amino acid	Pediatric and young adult patients (2–25 years old) with refractory SSTR-positive solid tumors	17	Renal, liver, and bone marrow dosimetry Toxicity	**Efficacy:** No CR reported, 2 patients showed PR, 5 patients showed minor responses, 6 patients experienced SD, 2 patients showed PD, and 2 patients withdrew **Safety:** Hyponatremia: Grade 4, IV electrolyte resuscitation, was reported in 1 patient Neutropenia: Grade 2 was reported in 2 patients Thrombocytopenia: Grade 2 was reported in 1 patient Grade 1 decrease in GFR was reported in 2 patients Carcinoid syndrome developed in 2 patients with metastatic NETs, which improved within 24 h after restarting octreotide No dose-limiting toxicities were reported No individual dose reductions due to renal or hematologic toxicity were reported
Phase 1, open-label, diagnostic study (NCT04040088) [60]	^68^Ga-DOTATATE	Pediatric and young adult patients (<30 years old) with NETs	Active, not recruiting	Difference in radiation treatment target volume definition between ^68^Ga-DOTATATE PET/CT and MIBG Proportion of agreement between ^68^Ga-DOTATATE PET/CT and MIBG Difference in metabolic activity between tumors diagnosed on ^68^Ga-DOTATATE PET/CT and MIBG. Pattern of failure after radiation therapy	Not published yet
**Studies of adult patients including some pediatric patients**					
Phase 2, open-label trial (NCT02236910) [61]	^177^Lu-DOTATATE	Patients, including pediatric patients (≥14 years), with SSTR-positive solid tumors	Active, not recruiting	Tumor response measured by RECIST criteria PFS QoL	Not published yet
Phase 1/2, open-label, diagnostic study (NCT03145857) [62]	^68^Ga-DOTATATE	Patients, including pediatric patients (≥14 years old), with SSTR-positive tumors	Recruiting	Changes in vital signs, hematology, and biochemistry after ^68^Ga-DOTATATE injection AEs Correlation of ^68^Ga-DOTATATE scan diagnostic effectiveness with standard of care CT or MRI Comparison ^68^Ga-DOTATATE scan vs. baseline scan	Not published yet
Phase 2 (NCT01237457) [63]	^177^Lu-DOTATATE	Patients, aged 11–87 years with inoperable, well-to-moderately differentiated, metastatic NETs	144	PFS OS Safety Toxicities	**Efficacy:** 28 patients completed therapy: no patient showed CR, 8 (29%) patients showed PR, 16 (57%) patients showed SD, and 4 (14%) patients showed PD; ORR (CR + PR) was 29% (*n* = 8); cumulative disease control (CR + PR + SD) was 86% (*n* = 24) 132 patients were evaluable for efficacy assessment: no patient showed CR, 12 (9%) patients showed PR, 66 (50%) patients showed SD, and 54 (41%) patients showed PD; ORR (CR + PR) was 9% (*n* = 12); and Cumulative disease control (CR + PR + SD) was 59% (*n* = 78) **Safety:** Abdominal pain, diarrhea, flushing, and fatigue improved in <50% of patients Weight loss improved in 28% of patients No Grade 3 or Grade 4 renal toxicities reported Hematologic AEs: Grade 3 and Grade 4 were reported in 11 and 5 patients, respectively, and lasted an average of 2.7 months and 0.9 months, respectively Hepatotoxicities: Grade 3 were reported in 4 patients and lasted on average 3.1 months
Phase 2, open-label, single-site trial (NCT01876771) [64]	^177^Lu-DOTATATE	Patients, aged 14–90 years with SSTR-positive NETs	Recruiting	Tumor response PFS Disease evaluation Change in tumor-marker level Safety Change in hematology, renal function, and liver function OS QoL	Not published yet

AE, adverse event; CI, confidence interval; CNS, central nervous system; CR, complete response; CT, computed tomography; CTCAE, Common Terminology Criteria for AEs; GEP, gastroenteropancreatic; GFR, glomerular filtration rate; IV, intravenous; MRI, magnetic resonance imaging; MTD, maximum tolerated dose; NET, neuroendocrine tumor; ORR, overall response rate; OS, overall survival; PD, progressive disease; PET, positron emission tomography; PFS, progression-free survival; PK, pharmacokinetics; PPGL, pheochromocytoma and paraganglioma; PR, partial response; QoL, quality of life; RECIST, Response Evaluation Criteria in Solid Tumors; RLT, radioligand therapy; RP2D, recommended phase 2 dose; SD, stable disease; SSTR, somatostatin receptor; SSTR-2A, SSTR subtype 2A; TRAE, treatment-related AE.

## Data Availability

No new data were created or analyzed in this study. Data sharing is not applicable to this article.

## Abbreviations

The following abbreviations are used in this manuscript:

NET	Neuroendocrine tumor
GEP-NET	Gastroenteropancreatic NET
PPGL	Pheochromocytoma and paraganglioma
^68^Ga-DOTATATE	^68^Ga-labeled DOTA-D-Phe^1^-Tyr^3^-Thr^8^-octreotate
^177^Lu-DOTATATE	[^177^Lu]Lu-DOTA-TATE
^111^In-DOTATATE	^111^In-DOTA-octreotate
^123/131^I-MIBG	^123/131^I-meta iodobenzyl-guanine
SSTR	Somatostatin receptor
RLT	Radioligand therapy
PET	Positron emission tomography
CNS	Central nervous system
CT	Computed tomography
^68^Ga-DOTATOC	^68^Ga-[tetraxetan-D-Phe^1^, Tyr^3^]-octreotide
^68^Ga-DOTANOC	[^68^Ga-DOTA,1-Nal^3^]-octreotide
FDA	U.S. Food and Drug Administration
EMA	European Medicines Agency
MRI	Magnetic resonance imaging
SPECT	Single-photon emission-CT
^18^F-FDG PET/CT	^18^F-fluorodeoxyglucose PET/CT
GD2	Ganglioside GD2
^90^Y-DOTATOC	^90^Y-DOTA^0^-Tyr^3^-octreotide
AE	Adverse event
PFS	Progression-free survival
IGF1	Insulin-like growth factor 1
SSA	Somatostatin analogs
GH	Growth hormone
